# Influence of Metal Interlayers on Spin-Charge Conversion in Sb_2_Te_3_ Topological Insulator-Based Devices

**DOI:** 10.1021/acs.nanolett.4c06658

**Published:** 2025-04-18

**Authors:** Emanuele Longo, Matteo Belli, Claudia Wiemer, Alessio Lamperti, Andrey V. Matetskiy, Polina M. Shevedyaeva, Paolo Moras, Marco Fanciulli, Roberto Mantovan

**Affiliations:** † Institut de Ciència de Materials de Barcelona (ICMAB-CSIC), Campus UAB, Bellaterra, Catalonia 08193, Spain; ‡ CNR-IMEM Unit of Parma, Parco area delle Scienze 37/A, 43124 Parma, Italy; § CNR-IMM, Unit of Agrate Brianza, Via C. Olivetti 2, 20864 Agrate Brianza, Italy; ∥ CNR-ISM Unit of Trieste, SS 14 Km 163,5, 34149 Trieste, Italy; ⊥ Department of Material Science, University of Milano Bicocca, Via R. Cozzi 55, Milan 20125, Italy

**Keywords:** spintronics, spin-charge conversion, topological insulators, ARPES, synchrotron

## Abstract

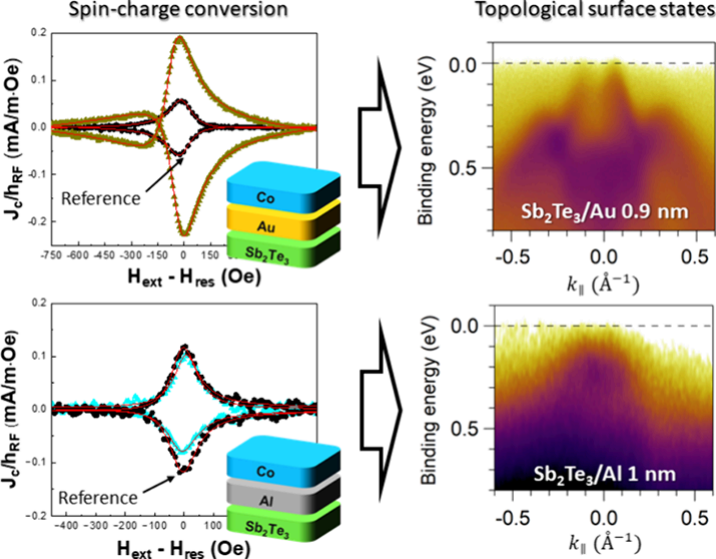

The magnetization of a ferromagnetic layer can be controlled via spin-charge conversion (SCC) phenomena originating in an adjacent topological insulator (TI). Insertion of nonmagnetic interlayers between these materials has been demonstrated to enhance the SCC efficiency, as shown for Sb_2_Te_3_/Au/Co(Fe) heterostructures. The inert nature of the Sb_2_Te_3_/Au interface was assumed to preserve the topological surface state (TSS) of Sb_2_Te_3_, which mediates the SCC. Al is explored as an alternative to Au for its long spin diffusion length. Spin pumping experiments indicate the absence of SCC in the Sb_2_Te_3_/Al/Co heterostructure. Core level and valence band photoemission spectroscopies reveal that Al forms stable compounds with Te and Sb, thereby quenching the TSS of Sb_2_Te_3_, while the TSS is preserved upon the formation of the TI/Au interface. These results demonstrate directly the major influence of material chemistry and highlight the role of TSS on the SCC efficiency in TI-based devices.

The functionality of electronic devices hinges on the intricate interplay between materials constituting the logic and memory components. Topological insulators (TIs) display promising properties for the development of fast, scalable and energy efficient devices.^1−3^ They are largely investigated in the context of spintronics, the branch of electronics which aims to exploit the spin angular momentum of the electron, in addition to the electron charge degree of freedom, to engineer new electronic device functionalities.^4−6^

The strength of TIs resides in their topological surface states (TSS).^2^ These states are robust against the introduction of nonmagnetic disorder (i.e., defects, impurities) and characterized by the so-called spin-momentum locking, which forces the spin and momentum directions of an electron flowing across the TSS to be mutually orthogonal in the reciprocal space. This property makes the TIs suitable for many applications based on magnetism in the fields of spintronics, magnonics, photonics and spin–orbitronics.^7−9^ A widely employed and promising strategy in this context is to exploit spin-charge conversion (SCC) mechanisms (i.e., the spin–orbit torque) originating in TIs to control the magnetization switching of adjacent ferromagnetic (FM) layers. Such mechanisms are considered highly efficient in terms of energy consumption with respect to inductive phenomena (i.e., Oersted effect)^6^ or the more recently studied spin-transfer torque.^9−11^

The use of nonmagnetic interlayers, which are transparent to spin currents and chemically separate the TIs from the FM layers, is known to produce efficient SCC devices.^12,13^ For example, previous studies revealed no SCC effect in the Sb_2_Te_3_/Co/Au heterostructure and high SCC efficiency upon the introduction of a Au interlayer at the Sb_2_Te_3_/(Co or Fe) interface.^14,15^ However, the role of the Sb_2_Te_3_ TSS on the observed behavior was not fully clarified. In fact, the SCC in TIs can arise from various mechanisms, including the bulk-related spin-Hall effect (SHE), as well as the Edelstein and Rashba effects, which are purely surface-driven processes.^16−18^

Motivated by previous work showing large room temperature (RT) SCC in the Si/Sb_2_Te_3_(30 nm)/Au/(Co or Fe)/Au systems,^14,15,19^ in the present study we explore the effect of using an Al interlayer in the Si/Sb_2_Te_3_(30 nm)/Al/Co/Au heterostructure. Al is chosen as a nonmagnetic material with longer spin diffusion length (*l*_*sd*_) than Au at RT (*l*_*sd*_^*Au*^ ∼ 35–60 nm, *l*_*sd*_^*Al*^ ∼ 300–600 nm), which, in principle, could impact positively on the SCC performances.^20,21^ Despite this expectation, spin pumping measurements show the absence of SCC, which we primarily ascribe to the quenching of the Sb_2_Te_3_ TSS by the strong chemical interaction with Al.

In order to shed light on the microscopic origin of the presence (absence) of SCC when using Au (Al) interlayers, we conducted a thorough photoemission spectroscopy study with synchrotron radiation on bulk Sb_2_Te_3_ single crystals. The effect of in situ depositions of Au and Al on the chemical composition and band structure of the Sb_2_Te_3_ surface was monitored by means of core level spectroscopy and angle-resolved photoemission spectroscopy (ARPES) measurements. As anticipated above, we observed the formation of stable Al–Te and Al–Sb alloys as minute amounts of Al (less than 1 nm) are deposited on freshly cleaved Sb_2_Te_3_, which lead to the disappearance of the TSS. Instead, Au turned out to be much less reactive with Sb_2_Te_3_, so that the TSS is preserved at the Au/Sb_2_Te_3_ interface. These experimental results highlight the role played by TSS in the definition of the SCC performances of the studied heterostructures. More in general, they stress the importance of using proper nonmagnetic interlayers to avoid the degradation of the chemical and electronic properties of the interfaces of TI-containing heterostructures. The reported fragility of TSS represents an obvious warning about the exploitation of TIs in future devices.

## Spin Pumping Ferromagnetic Resonance in Sb_2_Te_3_/Au/Co/Au and Sb_2_Te_3_/Al/Co/Au Heterostructures

**Figure 1:**
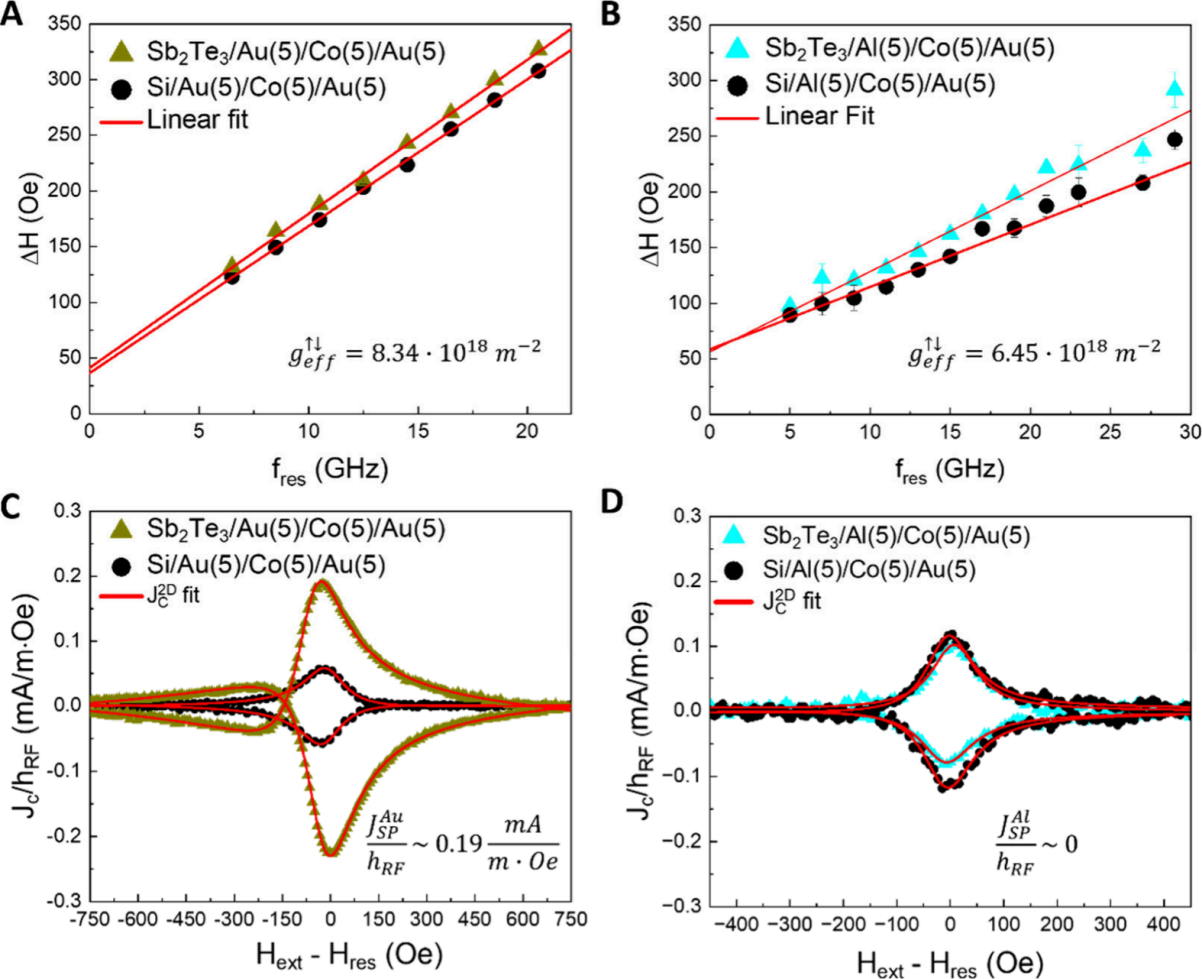
Spin pumping measurements on the Sb_2_Te_3_/Au and Sb_2_Te_3_/Al based systems. (A) and (B) compare the Δ*H*(*f*_*res*_) curves obtained for the Sb_2_Te_3_/Au/Co (green triangles) and Sb_2_Te_3_/Al/Co (light blue triangles) heterostructures against their respective Sb_2_Te_3_-free reference structures (black circles). Panels (C) and (D) show the SP-FMR signals measured for the same heterostructures. The red solid lines represent fits to the data using eq 3 (described in Materials and Methods in the Supporting Information), which were used to extract and compare the normalized 2D charge current density (*J*_*C*_^2D^/*h*_*RF*_) values across the different systems. The numbers in brackets indicate the nominal thickness in nm.

Figure 1 shows the broadband ferromagnetic resonance (BFMR) and spin pumping FMR (SP-FMR) measurements conducted on the Si/Sb_2_Te_3_(30)/Au(5)/Co(5)/Au(5) and Si/Sb_2_Te_3_(30)/Al(5)/Co(5)/Au(5) heterostructures, and on their corresponding reference systems without Sb_2_Te_3_ (Si/Au(5)/Co(5)/Au(5) and Si/Al(5)/Co(5)/Au(5)). The number in brackets indicates the layer thickness expressed in nm. According to the analysis of the Kittel dispersion, the magnetodynamic properties of the deposited Co thin films (i.e., g-factor, effective magnetization) are consistent with those of polycrystalline Co, thus making both samples a reliable platform to study SCC effects (see Figure S1).

In Figure 1A and 1B, the line width (Δ*H*) of the resonance curves as a function of the resonant frequency (*f*_*res*_ is shown for the samples described above. The acquired data sets are fitted with the following equation:
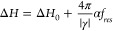
1where  is the gyromagnetic ratio (*g* is the g-factor, *e* the electron charge and *m*_*e*_ the effective mass for the free electron), Δ*H*_0_ indicates the inhomogeneous broadening (i.e., full width at half-maximum) accounting for the magneto-structural disorder of the FM film (i.e., structural disorder, magnetic dead layers) and α is the damping constant. For the samples with the Au interlayer (Figure 1A), the intercept Δ*H*_0_ is the same within the error bar for both the structures, with and without the Sb_2_Te_3_ layer (Δ*H*_0_ = 39 ± 6 *Oe*). Similarly, the samples with the Al interlayer show the same inhomogeneous broadening (Δ*H*_0_ = 57 ± 7 *Oe*) (Figure 1B), indicating that the deposited Co layers possess the same magneto-structural properties. Furthermore, in the presence of Sb_2_Te_3_ a clear increase of the Δ*H*(*f*_*res*_) curves slope emerges, a signature of the enhanced α value, in agreement with eq 1. The fitted values for α are α_*Sb*_2_*Te*_3__^*Au*^ = (25.5 ± 0.6) × 10^–3^, α_*Si*_^*Au*^ = (20.3 ± 0.2) × 10^–3^, α_*Sb*_2_*Te*_3__^*Al*^ = (11.2 ± 0.5) × 10^–3^ and α_*Si*_^*Al*^ = (9.2 ± 0.5) × 10^–3^.

According to the SP theory, the enhancement of α relates to the real part of the so-called spin mixing conductance (*g*_*eff*_^↑↓^), an intrinsic quantity which accounts for the spin-transparency of a specific nonmagnetic/ferromagnetic (NM/FM) material interface and expressed as
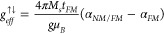
2where *μ*_*B*_ is the Bohr magneton and *t*_*FM*_ the thickness of the FM layer. By using eq 2 we calculate *g*_*eff,Au*_^↑↓^ = 8.34 × 10^18^ m^–2^ and *g*_*eff,Al*_^↑↓^ = 6.45 × 10^18^ m^–2^, which are compatible with the values extracted from similar topological systems (see Tab. One in ref.^15^). The *g*_*eff*_^↑↓^ values calculated for the Sb_2_Te_3_/Au/Co and Sb_2_Te_3_/Al/Co are comparable, indicating that the use of the Al interlayer to generate spin currents in the Co layer can be, in principle, almost equivalent to the Au one.

More specifically, being *g*_*eff*_^↑↓^ proportional to the 3D spin current (*J*_*S*_^3*D*^) generated in the FM layer, the *g*_*eff*_^↑↓^ value observed in the Al-based heterostructure indicates that a significant *J*_*S*_^3*D*^ is generated in Co due to the presence of the Sb_2_Te_3_ spin sink layer. Nevertheless, it is now well accepted that the observation of the α value enhancement may not be necessarily connected to the occurrence of SCC.^14^

In Figure 1C and 1D the electrically detected SP-FMR signals acquired for Sb_2_Te_3_/Au(5)/Co(5)/Au(5) (green triangles) and Si/Au(5)/Co(5)/Au(5) (black circles) and for Sb_2_Te_3_/Al(5)/Co(5)/Au(5) (light blue triangles) and Si/Al(5)/Co(5)/Au(5) (black circles) are reported, for *f*_*res*_ = 10.5 *GHz* and *f*_*res*_ = 11 *GHz*, respectively. In both panels, the *y*-axis corresponds to the 2D charge current density (*J*_*C*_^3*D*^) normalized over the magnitude of the exciting oscillating magnetic field (*h*_*RF*_), while the *x*-axis represents the shift of the applied external magnetic field from the resonance field (*H*_*ext*_ - *H*_*res*_). Here, the red solid lines indicate the fit with eq 3 reported in the Materials and Methods section. For further details on the SP-FMR experimental technique please refer to ref.^15^

The electrically detected SP-FMR signals are positioned around the same *H*_*res*_ value, as measured by BFMR for the same *f*_*res*_, thus demonstrating that *J*_*C*_^2*D*^ is originated from the magnetization dynamics induced in the Co layer (see Figure S1 A and B).

In Figure 1C, the magnitude of the two *J*_*C*_^2*D*^/*h*_*RF*_ curves where the Au interlayer is adopted is remarkably enhanced for the structure with Sb_2_Te_3_ with respect to the reference. As detailed in ref.,^15^ by extracting the spin pumping component of the *J*_*C*_^2*D*^/*h*_*RF*_ curve in the presence of Sb_2_Te_3_ (green triangles in Figure 1C), we find  mA/(m·Oe). Such a value is 3.5 times larger than the one extracted for the Sb_2_Te_3_-free reference (black circles), being  mA/(m·Oe). This result demonstrates that the Sb_2_Te_3_/Au/Co based heterostructures are suitable for developing highly efficient SCC devices.^15^

Conversely, when the Al interlayer is adopted as spacer at the Sb_2_Te_3_/Co interface, the magnitude of the two *J*_*C*_^2*D*^/*h*_*RF*_ curves is barely the same (Figure 1D), indicating that the introduction of the Sb_2_Te_3_ layer does not generate an enhancement of the SCC efficiency. The minor discrepancies observed between the curves with and without the Sb_2_Te_3_ layer, as shown in Figure 1D, can be ascribed to subtle variations in the crystalline quality of the materials and/or differences in thermal contributions, such as the Seebeck effect.^14,15^ These findings support the conclusion that no SCC is detected in the Sb_2_Te_3_/Al/Co/Au systems under the investigated conditions.

## Photoemission Spectroscopy Study of the Sb_2_Te_3_/Au and Sb_2_Te_3_/Al Interfaces

In order to determine the microscopic origin of the different behavior associated with the presence of Al or Au spacers, we performed in situ photoemission spectroscopy measurements of Al and Au deposits on freshly cleaved bulk Sb_2_Te_3_ (see Materials and Methods).

**Figure 2:**
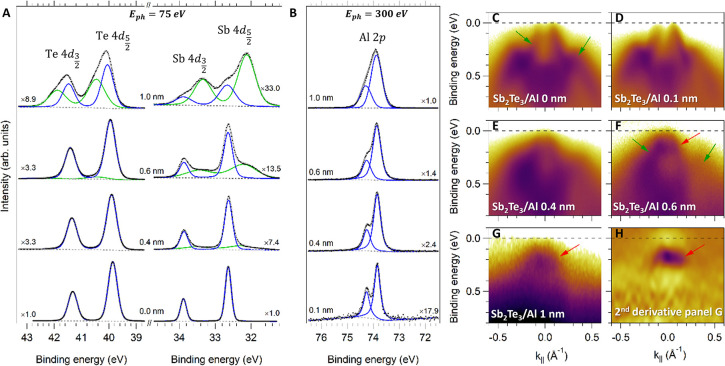
Photoemission spectroscopy measurements on the Sb_2_Te_3_/Al samples as a function of the Al thickness. (A) shows the evolution of the Te 4d and Sb 4d peaks as a function of the Al thickness for E_ph_ = 75 eV. A clear modification of the signals shape is observed in the studied thickness range. (B) Al 2p peaks as a function of the Al thickness acquired at E_ph_ = 300 eV. The well-resolved Al doublet present at *t* = 0.1 nm progressively transforms into a broad peak due to the interaction with Sb_2_Te_3_. For better comparison, the intensity is multiplied by a factor shown close to each curve. (C)–(G) In situ ARPES measurements on the Sb_2_Te_3_(bulk)/Al heterostructure as a function of the Al thickness (E_ph_ = 75 eV). The deposition of Al has a significant impact on the band dispersion in the proximity of the Fermi level, leading to a gradual disappearance of TSS when the thickness reaches 0.6 nm, and the appearance of a different electronic pattern. The intensity is shown on a logarithmic scale. (H) Second derivative of the signal in panel (G). Green arrows mark the TSS and the bulk states of Sb_2_Te_3_, red arrow indicates the new electronic feature.

Figure 2A displays the Te 4d and Sb 4d core level spectra obtained from the Sb_2_Te_3_(bulk)/Al(t) (t = 0, 0.4, 0.6, 1.0 nm indicate the nominal thickness of the Al layer) samples for an incident photon energy (E_ph_) of 75 eV. The Te 4d_5/2_ (Sb 4d_5/2_) peak of clean Sb_2_Te_3_ is located at 39.8 eV (32.5 eV), in agreement with the literature.^22^ As expected, when the Al thickness increases, the intensity of the Te and Sb 4d peaks is reduced, as accounted by the multiplying factors reported near the spectra. Correspondingly, the Al 2p signal in Figure 2B (E_ph_ = 300 eV) increases. From 0.4 nm Al thickness, the Sb signal shows the formation of an additional doublet having Sb 4d_5/2_ at 32.1 eV, corresponding to metallic Sb.^22^ In line with recent reports,^23^ this can be associated with the formation of clusters of metallic Sb, which migrates from the interface up to the surface due to its surfactant nature. Furthermore, a pronounced broadening of the original Sb 4d doublet is observed at 1.0 nm thickness. A shift to higher binding energies of the original Te 4d doublet by 0.2 eV and the appearance of an extra doublet (Te 4d_5/2_ peak at 40.5 eV) is clearly seen at 1.0 nm Al thickness. The position of the Al 2p_3/2_ peak (73.8 eV) is found at higher values with respect to pure metallic Al (Al 2p_3/2_ at 73.0 eV) already at 0.1 nm thickness. All these observations can be associated with the formation of Al–Sb and/or Al–Te based compounds immediately after the Al deposition.^24^ By following thermodynamic considerations, Al tends to form stable AlSb and Al_2_Te_3_ binary phases when mixed up with Sb_2_Te_3_.^25^ As detailed in the Supporting Information, such reactions possess negative standard-state Gibbs free energy (Δ*G*_*Compound*_^*o*^) variation, and, therefore, represent energetically stable thermodynamic processes. Notably, the formation of Al–Te and Al–Sb alloys has been recently reported in Al-covered Bi_0.12_Sb_1.88_Te_3_, which is closely related to Sb_2_Te_3_.^26^

In panels C - H of Figure 2, the ARPES measurements acquired on the Sb_2_Te_3_(bulk)/Al(t) samples around the Γ point (*k*_∥_= 0 Å^–1^) of the Sb_2_Te_3_ surface Brillouin zone are shown (E_ph_ = 75 eV). The ARPES measurements were performed in the very same sample spot and geometrical configuration of the core level measurements discussed above. Panel C of Figure 2 shows the presence of TSS on the bare Sb_2_Te_3_ materials, in the form of two linearly dispersing bands which meet at the Dirac point just above the Fermi level (leftmost green arrow). This feature gives a p-type character to the Sb_2_Te_3_ conduction, as previously observed for pure Sb_2_Te_3_ single crystals^27−30^ and predicted by theoretical calculations.^27^ The features below 0.3 eV (rightmost green arrow) are bulk states of Sb_2_Te_3_. In panels D to G, we observe the progressive disappearance of the TSS and bulk states of Sb_2_Te_3_, gradually replaced by new electronic features at about 0.2 eV (red arrow) that are better visualized in panel H, where the second derivative of the signal reported in panel G is shown. The dispersion of these states is significantly different from the original electronic structure of Sb_2_Te_3_, indicating the occurrence of a strong chemical change at the interface region. Since ordered patches of Al–Sb have been reported to form in Al-covered Bi_0.12_Sb_1.88_Te_3_,^26^ we tentatively associate the new electronic states to this material. Notably, no remnants of TSS and bulk states of the original Sb_2_Te_3_ substrate can be observed at 1.0 nm Al thickness.

**Figure 3:**
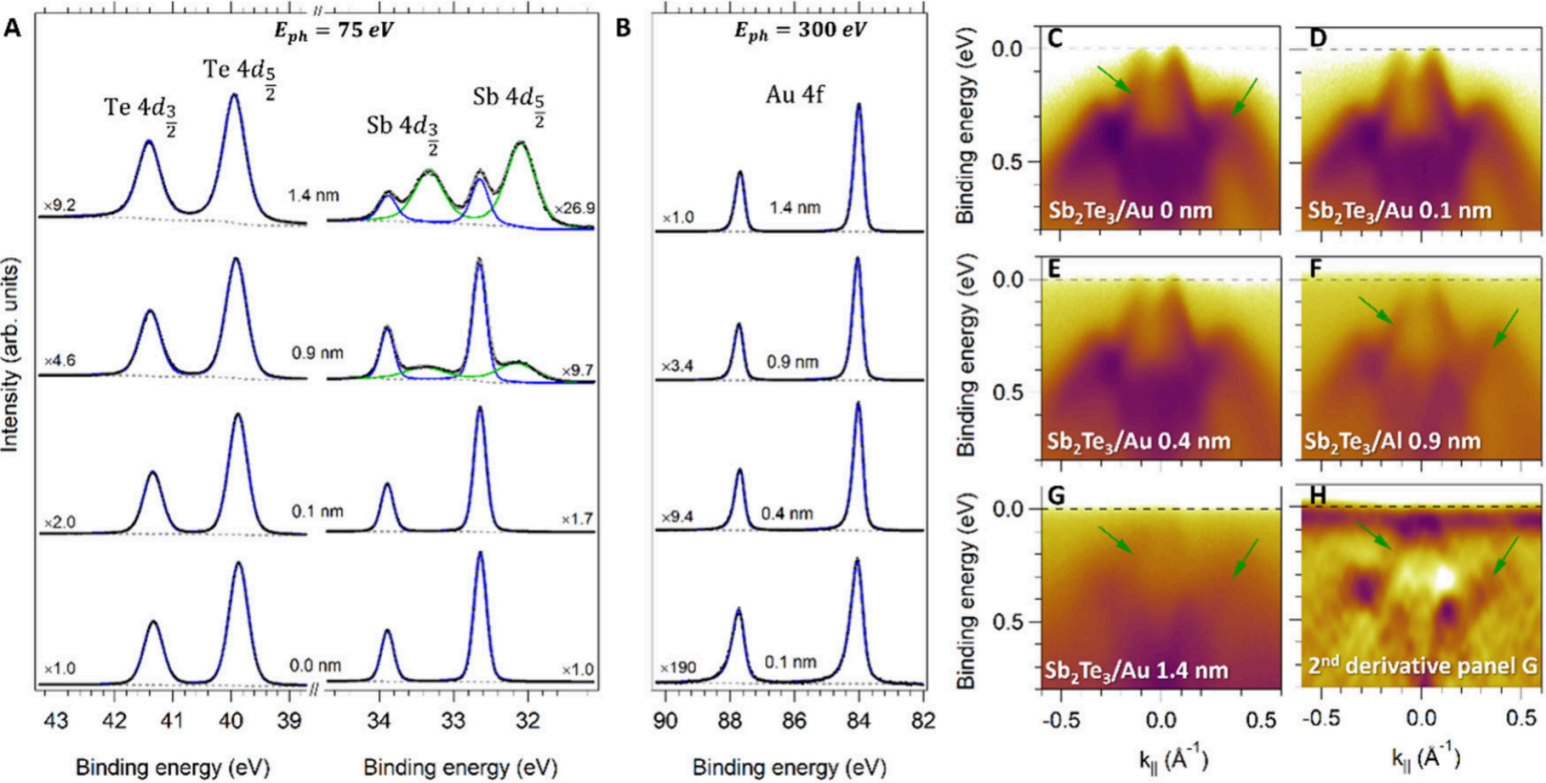
Photoemission spectroscopy measurements on the Sb_2_Te_3_/Au samples as a function of the Au thickness. (A) shows the evolution of the Te 4d and Sb 4d peaks as a function of the Au thickness for E_in_ = 75 eV. A modification of the Sb 4d states is observed for the Au thickness around 0.9 nm. (B) Au 4f peaks as a function of the Al thickness acquired at E_in_ = 300 eV. The Au doublet maintains the same shape and position as a function of the Au thickness, demonstrating the negligible chemical interaction between the Au atoms and the Sb_2_Te_3_ crystal. For better comparison, the intensity is multiplied by a factor shown close to each curve. (C)-(G) In situ ARPES measurements on the Sb_2_Te_3_(bulk)/Au heterostructure as a function of the Au thickness. The uniform signal background originated by the Au deposition is clearly visible in panel (H) for Au = 1.4 nm. The black horizontal dashed lines indicate the position of the Fermi level. The intensity is shown on a logarithmic scale. (H) Second derivative of the signal in panel (G). Green arrows mark the TSS and the bulk states of Sb_2_Te_3_.

To study the chemical status of the Sb_2_Te_3_/Au interface, core level and ARPES measurements are conducted similarly to what performed for the Sb_2_Te_3_/Al sample. Figure 3A displays the Te 4d and Sb 4d core levels acquired at E_ph_ = 75 eV as a function of the nominal thickness of the Au layer (t = 0, 0.1, 0.9, 1.4 nm). Here, the reduction of the Te and Sb peaks intensity is accounted for by the multiplying factor reported near the spectra. It is important to note that the formation of Sb metallic clusters also occurs in the Au case, as evidenced by the additional doublet with Sb 4d_5/2_ at 32.1 eV emerging upon the deposition of 0.9 and 1.4 nm of Au.^23^ Unlike the Al case, the Te signal is marginally modified by the presence of Au, besides a peak broadening and a small shift of 0.05 eV to higher binding energies at 1.4 nm Au thickness.^23^ The Au 4f metallic core levels (Au 4f_7/2_ at 84.0 eV) are well-defined doublets, confirming the robust chemical stability of the Sb_2_Te_3_/Au interface. According to thermodynamic considerations, the most probable compounds forming between Au and Sb_2_Te_3_ are AuTe_2_ and AuSb_2_, for which Δ*G*_*Compound*_^*o*^ assumes positive values, thus indicating that the chemical intermixing is not spontaneous at RT (see Supporting Information).

The low reactivity of Au with Sb_2_Te_3_ is consistent with similar observations on other Au/TI interfaces^31^ and in line with the predicted negligible hybridization between Au states and TSS, thus preserving their spin-momentum locking topological feature.^32^ Panels C–H of Figure 3 represent the ARPES spectra as obtained from the Sb_2_Te_3_(bulk)/Au(t) set acquired around the Γ point of the Sb_2_Te_3_ surface Brillouin zone, on the same sample spot of the core level experiments. Here, Au depositions do not affect the shape of the TSS and bulk states of Sb_2_Te_3_ (green arrows) up to 1.4 nm, at variance with the Al case. It is worth noting that, at the used photon energy, the mean free path of the photoelectrons is approximately 0.5 nm.^33^ As a result, at an Au thickness of 1.4 nm, the photoelectron intensity from the Sb_2_Te_3_ substrate is attenuated by a factor of about 20. Despite the significant increase of the background signal, Sb_2_Te_3_-related features remain visible, as shown in panels G and H (second derivative of panel G) of Figure 3. Notably, no new electronic features emerge upon Au deposition, in analogy with the Bi_2_Se_3_/Au case.^34^

The spectroscopic analysis demonstrates the primary role of interface material chemistry in the Sb_2_Te_3_/Al and Sb_2_Te_3_/Au systems and, correspondingly, in the heterostructures containing Al or Au interlayers. The formation of Al–Sb and Al–Te alloys has detrimental effects (quenching) on the TSS, while the bulk states are certainly preserved in the Sb_2_Te_3_ material away from the interface region. For this reason, the absence of SCC in the Sb_2_Te_3_/Al/Co/Au heterostructure is linked to the absence of the TSS. Instead, the well-defined structural and electronic properties of the Sb_2_Te_3_/Au interface led to enhanced SCC in the Sb_2_Te_3_/Au/Co/Au heterostructure. In general, these results strongly suggest that the Inverse Edelstein effect^15^ mainly determines the SCC in TI-based heterostructure, although contributions from bulk-related effects cannot be excluded.

In this manuscript the effect of Au and Al interlayers in the Sb_2_Te_3_/Au/Co/Au and Sb_2_Te_3_/Al/Co/Au systems is investigated by means of BFMR and SP-FMR techniques. Despite BFMR having proved the possibility to generate spin currents when the Al interlayer is adopted, no SCC was observed. To elucidate the origin of the missing SCC effect, ARPES and core level measurements were employed to study the chemical intermixing at the interface between a bulk Sb_2_Te_3_ single crystals and Al layers of different thicknesses. The deposition of a 1.0 nm thick Al layer quenched the Sb_2_Te_3_ TSS, which is thus coherent with the absence of TSS-mediated SCC. Conversely, the Sb_2_Te_3_ TSS turned out to be almost unperturbed upon the Au deposition.

This work addresses several critical open questions about spintronic systems based on TIs and their TSS (see the Supporting Information for further discussion). First, the results highlight the importance of choosing a suitable nonmagnetic interlayer for preserving TSS, since its chemical reactivity may lead to the disappearance of these states. Second, it was previously unclear whether TSS, once disrupted in the topmost layer of a topological material due to chemical intermixing, could reform at a new interface beneath the intermixed region. This study shows that such restoration does not occur in the Sb_2_Te_3_/Al interface. Lastly, ARPES measurements point out the role of the Inverse Edelstein effect in the SCC performances of the Sb_2_Te_3_/Au/Co/Au heterostructure.^15^ The comparison of SP-FMR, ARPES, and core level measurements presented in this manuscript offers valuable insights for advancing the application of Sb_2_Te_3_ and, more broadly, chalcogenide TIs materials in spintronic devices leveraging SCC effects.

## Supporting Information Available

Additional experimental details, materials, and methods, Kittel curve for Sb_2_Te_3_/Au/Co/Au and Sb_2_Te_3_/Al/Co/Au heterostructures, thermodynamic considerations on Al and Au alloys formation with Sb_2_Te_3_ at RT, Te core levels attenuation for different Au depositions, and discussion on Sb_2_Te_3_/Co interface spin current transparency and role of TSS in SCC (PDF)
