# Supplementary material for: Influence of Metal Interlayers on Spin-Charge Conversion in Sb2Te3 Topological Insulator-Based Devices
Source: Nano Lett. 2025 Apr 18;25(17):6888–94. doi: 10.1021/acs.nanolett.4c06658 (PMC12046589; doi:10.1021/acs.nanolett.4c06658)
Supplement: Supplementary file 1 — nl4c06658_si_001.pdf [file nl4c06658_si_001.pdf]

Influence of Metal Interlayers on Spin-Charge Conversion in Sb_2Te_3 Topological Insulator-Based Devices

EMANUELE LONGO[1*], MATTEO BELLI[2], CLAUDIA WIEMER[3], ALESSIO LAMPERTI[3], ANDREY V. MATETSKIY[4], POLINA M. SHEVEDYAEVA[4], PAOLO MORAS[4], MARCO FANCIULLI[5], ROBERTO MANTOVAN[3*]

1. Institut de Ciència de Materials de Barcelona (ICMAB-CSIC), Campus UAB, Bellaterra, Catalonia 08193, Spain
2. CNR-IMEM Unit of Parma, Parco area delle Scienze 37/A, 43124 Parma, Italy
3. CNR-IMM, Unit of Agrate Brianza, Via C. Olivetti 2, 20864 Agrate Brianza, Italy
4. CNR-ISM Unit of Trieste, SS 14 Km 163,5, 34149 Trieste, Italy
5. Department of Material Science, University of Milano Bicocca, Via R. Cozzi 55, Milan 20125, Italy

*elongo@icmab.es *roberto.mantovan@cnr.it

Keywords: spintronics, spin-charge conversion, topological insulators, ARPES, synchrotron

Supplementary Information

1. Materials and Methods

Samples probed by ferromagnetic resonance techniques were prepared by Metal Organic Chemical Vapor deposition on i-Si(111) 4'' Si wafers. Further details concerning the growth methods can be found in Ref.[19] of the main text. Al and Au depositions on bulk Sb_2Te_3 for ARPES and core level analysis were performed by thermal evaporation under ultra-high vacuum conditions in the growth chamber of the VUV-Photoemission beamline. The nominal thickness of the Al layers was defined on the basis of the deposition time from an Al source, whose evaporation rate was calibrated through a thickness monitor (estimated error of ±15%). The nominal thickness of the Au layers was determined by tracking the extinction of the Te signal at photon energy 300 eV, as detailed in Section 4 (estimated error of ±15%).

The bulk Sb_2Te_3 material was a natural single crystal 99,9999% pure purchased from the *2D semiconductor* company.

BFMR is performed using a broadband Anritsu-MG3694C power source (1-40 GHz), connected to a grounded coplanar waveguide, where the samples are mounted in a flip-chip configuration (the FM film is located close to the GCPW surface), with a 75 μm thick Kapton foil stacked in between to prevent the shortening of the conduction line. The sample-GCPW system is positioned between the polar extensions of a Bruker ER-200 electromagnet

maintaining its surface parallel to the external magnetic field H_{ext}, in the so-called in-plane (IP) configuration. During the measurements, an RF current at a fixed frequency is carried toward the GCPW and the transmitted signal is directed to a rectifying diode, converting the RF-signal into a continuous DC-current, subsequently detected by a lock-in amplifier downwards the electronic line. The same instrumentation is adopted to conduct SP-FMR measurements. Here, the edges of the sample are contacted with Ag paint and connected to a nanovoltmeter. A DC-voltage is detected in resonant condition, fixing the RF frequency and power.

The electrical signal reported in Fig. 1C is fitted with the following equation:

$$\frac{J_{mix}}{h_{RF}} = \frac{V_{mix}}{WRh_{RF}} = \frac{1}{WRh_{RF}}\left[V_{Sym}\frac{\Delta H^2}{\Delta H^2+(H-H_{res})^2} + V_{Asym}\frac{\Delta H(H-H_{res})}{\Delta H^2+(H-H_{res})^2}\right] \quad (3)$$

where, W and R represent the width of the heterostructure and its sheet resistance, respectively. V_{Sym} and V_{Asym} are the symmetric and the anti-symmetric component of the Lorentzian curves.

The photoemission experiments were performed at the VUV-Photoemission beamline at Elettra synchrotron in Trieste, Italy. The bulk Sb_2Te_3 was cleaved in ultra-high vacuum conditions to expose the (00ℓ) fresh surface and Al and Au layers of variable thickness were deposited on top of it. Core level photoemission experiments were carried out to follow the evolution of the Al 2p, Au 4f, Sb 4d and Te 4d core levels. ARPES measurements of the valence band were carried out with 75 eV photon energy (E_{ph}). ARPES and core level photoemission experiments were performed on the very same sample spot (spot size 300 × 100 μm^2).

2. Kittel curve for Sb_2Te_3/Au/Co/Au and Sb_2Te_3/Al/Co/Au heterostructures

According to the Landau-Lifshitz-Gilbert model for the dynamics of the magnetization vector in a ferromagnetic material, when an external magnetic field is applied in the film plane (IP), the evolution of the resonant frequency (f_{res}) of the magnetization vector as a function of the resonant magnet field (H_{res}) can be expressed through the Kittel equation for the IP configuration (Eq. 1).

$$f_{res} = \frac{\gamma}{2\pi}\sqrt{H_{res}\,(H_{res} + 4\pi M_{eff})} \qquad (1)$$

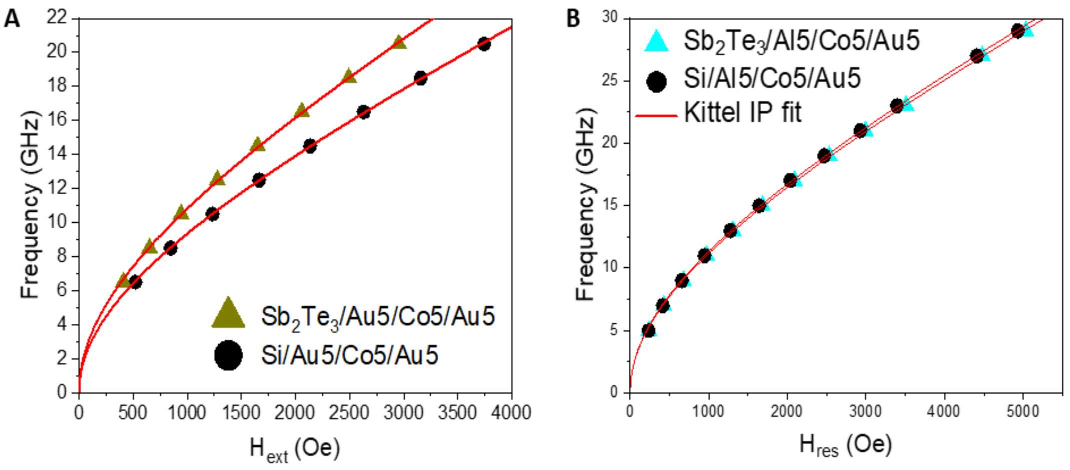

Fig. S1. In panel (A) and (B) the Kittel dispersion for the Sb_2Te_3/Au/Co/Au and Sb_2Te_3/Al/Co/Au heterostructures are reported, respectively.

where $\gamma = g\,\frac{e}{2m_e}$ is the gyromagnetic ratio (g is the g-factor, e the electron charge and m_e the effective mass for the free electron) and M_{eff} is the effective magnetization, to account for the surface magnetic anysotropy present in the Co film. In Fig. S1A the Kittel evolution for the two samples is reported for Sb_2Te_3/Au(5)/Co(5)/Au(5) (green triangles) and Si/Au(5)/Co(5)/Au(5) (black circles). Similarly, in Fig. S1B the Kittel dispersion is reported for the Sb_2Te_3/Al(5)/Co(5)/Au(5) (light blue triangles) and Si/Al(5)/Co(5)/Au(5) (black circles) systems. The datasets are fitted with Eq. 1 and the parameter of interest are extracted. For the samples with Au interlayer the fit yields $\gamma_{Sb2Te3}^{Au} = (2.32 \pm 0.70) \cdot 10^7 Hz/Oe$, $\gamma_{Si}^{Au} = (1.93 \pm 0.33) \cdot 10^7 Hz/Oe$, $M_{eff}^{Au,Sb_2Te_3} = 603 \pm 46\ emu/cm^3$ and $M_{eff}^{Au,Si(111)} = 653 \pm 29\ emu/cm^3$. In the case of Al the extracted parameters are $\gamma_{Sb2Te3}^{Al} = (2.07 \pm 0.40) \cdot 10^7 Hz/Oe$, $\gamma_{Si}^{Al} = (2.07 \pm 0.43) \cdot 10^7 Hz/Oe$, $M_{eff}^{Al,Sb_2Te_3} = 839 \pm 42\ emu/cm^3$ and $M_{eff}^{Al,Si(111)} = 861 \pm 46\ emu/cm^3$. The extracted γ values for all the heterostructures are compatible with those reported for Co thin films. Moreover, M_{eff} turned out to be the same for the samples with Sb_2Te_3 and their corresponding references, within the experimental uncertainties, indicating that the magnetic properties of the evaporated Co layers are driven by the contact with the Au and Al layers as expected.

3. Thermodynamic consideration on Al and Au alloys formation with Sb_2Te_3 at RT

The most probable reactions taking place between the Al atoms and the Sb_2Te_3 are restricted to the AlSb and the Al_2Te_3 binary phases. To evaluate degree of spontaneity of such reactions, the standard-state Gibbs free energy variation $\Delta G^o_{Compound}(\Delta H^0, \Delta S^0)$ as a function of the changes in the enthalpy ΔH^0 and entropy ΔS^0 is calculated for a system at RT. The adopted equation is $\Delta G^o_{Compound} = \Delta H^0 - T\Delta S^0$, and the parameters of interest are extracted from the tabulated values reported in Ref.[1]. The chemical reactions considered for the AlSb (R1) and the Al_2Te_3 (R2) formation are

$$Sb_2Te_3 + 2Al \rightarrow 2AlSb + 3Te \qquad \Delta G^0_{AlSb} = -70.1 \frac{kJ}{mol{\cdot}Al} \qquad [R1]$$

$$Sb_2Te_3 + 2Al \rightarrow 2Al_2Te_3 + 2Sb \qquad \Delta G^0_{Al2Te3} = -127.6 \frac{kJ}{mol{\cdot}Al}. \qquad [R2]$$

The extracted negative values $\Delta G^0_{AlSb} = -70.1 \frac{kJ}{mol{\cdot}Al}$ and $\Delta G^0_{Al2Te3} = -127.6 \frac{kJ}{mol{\cdot}Al}$ for the AlSb and Al_2Te_3 compounds, respectively, shows that both the reactions are energetically favored during the growth, being the Al_2Te_3 formation the most likely event.

For the Au case, the most probable compounds forming upon Au deposition on Sb_2Te_3 are $AuTe_2$ and $AuSb_2$. The corresponding chemical reactions are reported in [R3] and [R4], respectively. Here, the calculated $\Delta G^o_{Compound}$ assumes positive values in both cases, indicating that the chemical intermixing is not spontaneous at RT, in accordance with the less pronounced modification of the core level signals (see Fig. 3A, B in the main text).

$$Sb_2Te_3 + 3Au \rightarrow 3AuTe_2 + 4Sb \qquad \Delta G^0_{AuTe_2} = 21.47 \frac{kJ}{mol{\cdot}Au} \qquad [R3]$$

$$Sb_2Te_3 + Au \rightarrow AuSb_2 + 3Te \qquad \Delta G^0_{AuSb_2} = 65.52 \frac{kJ}{mol{\cdot}Au}. \qquad [R4]$$

4. Te core levels attenuation for different Au depositions

Fig. 3 of the main text shows that the interface reactivity of the Au/Sb_2Te_3 system is much lower than that of Al/Sb_2Te_3, besides the observed migration of Sb to the surface. Assuming that the Au growth is sufficiently flat, it is possible to determine the nominal thickness t of

the Au layers from the attenuation of the Te core level signal, since Te is marginally involved in the formation of new compounds. Figure S2 represents the area of the Te 4d peaks taken with E_{ph}= 300 eV as a function of the Au deposition time. The decay is fitted by using the formula $A_0 \cdot e^{-DT/\lambda}$, where A_0 is the initial Te signal (set to 1), λ is the inelastic mean free path of (photo)electrons crossing the Au layer, D is the evaporation rate and T the deposition time (t = DT). At 260 eV (i.e. the kinetic energy of Te 4d at E_{ph}= 300 eV) λ=0.86 nm.[2] From the fit we derive D = 0.017 nm/min, which provides the values of the nominal thickness. In Figure S2 the experimental points corresponding to the thinnest layers present sizable deviation from the fitted curve, which translates into an estimated error of ±15%.

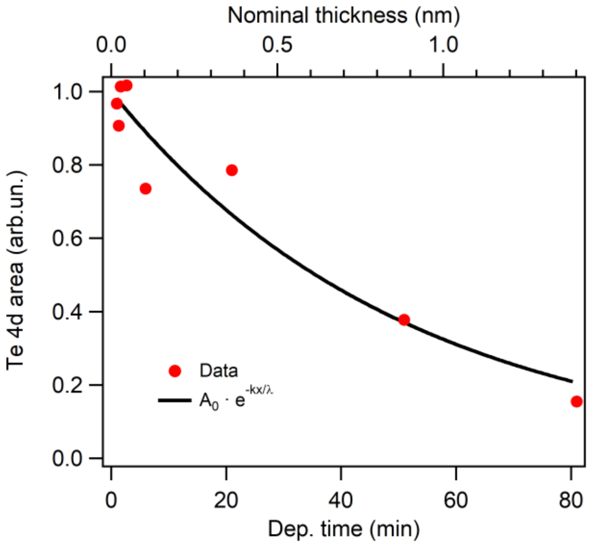

Fig. S2. Area of the peak of the Te 4d core levels as a function of the Au deposition time. The black line represents the fit to the experimental data.

6. Discussion on Sb_2Te_3/Co interface spin current transparency and role of TSS in SCC

To further highlight the role of TSS in SCC and the impact of interface quality, we provide additional discussion based on prior studies and the present experimental findings.

We linked the absence of SCC in the Sb_2Te_3/Co heterostructures mainly to TSS quenching. However, interface effects such as reduced spin transparency or spin memory loss must also be considered. This was previously investigated in Sb_2Te_3/Fe and Sb_2Te_3/Au/Fe heterostructures combining Mössbauer spectroscopy, X-ray reflectivity and spin pumping measurements.[3] Although Fe layers displayed comparable quality in the two systems, SCC

was observed only in the structure with the Au interlayer, indicating that direct interaction between Sb_2Te_3 and Fe atoms suppresses SCC. This suggests that a similar mechanism could be present in Sb_2Te_3/Co, reinforcing the fact that the spin memory loss mechanism alone is unlikely to explain the absence of SCC.[4] Additionally, our spin mixing conductance measurements for the Sb_2Te_3/Au/Co and Sb_2Te_3/Al/Co heterostructures studied in the main text show comparable values (i.e., $g^{\uparrow\downarrow}_{eff,Au} = 8.34 \cdot 10^{18}\ m^{-2}$ and $g^{\uparrow\downarrow}_{eff,Al} = 6.45 \cdot 10^{18}\ m^{-2}$), confirming similar spin transparency. Furthermore, previous reports in similar topological insulator/ferromagnet systems, indicate that Co deposition on Sb_2Te_3 strongly modifies the TSS of TIs, aligning well with our evidence of SCC suppression in Sb_2Te_3-based systems.[5] These findings further support that the heterostructures studied in this work exhibit comparable spin current dynamics, reinforcing the role of TSS in SCC.

References

1. Barin, I. Thermochemical Data of Pure Substances. *Thermochemical Data of Pure Substances* (1995) doi:10.1002/9783527619825.
2. Seah, M. P. & Dench, W. A. Quantitative electron spectroscopy of surfaces: A standard data base for electron inelastic mean free paths in solids. *Surface and Interface Analysis* **1**, 2–11 (1979).
3. Longo, E. *et al.* Spin-Charge Conversion in Fe/Au/Sb 2 Te 3 Heterostructures as Probed By Spin Pumping Ferromagnetic Resonance. *Adv Mater Interfaces* **2101244**, 2101244 (2021).
4. Longo, E. *et al.* Large Spin-to-Charge Conversion at Room Temperature in Extended Epitaxial Sb2Te3 Topological Insulator Chemically Grown on Silicon. *Adv Funct Mater* **2109361**, (2021).
5. Kaveev, A. K. *et al.* Band gap opening in the BiSbTeSe2 topological surface state induced by ferromagnetic surface reordering. *Phys Rev Mater* **5**, (2021).
